# Agentic AI in global health equity for high altitude populations

**DOI:** 10.1038/s41746-026-02701-7

**Published:** 2026-06-05

**Authors:** Peilun Shi, Tianmin Ren, Yuan Xie, Judy Wawira Gichoya, Tien Yin Wong, Xunming Ji, Ningli Wang, Wu Yuan

**Affiliations:** 1 Department of Biomedical Engineering, The Chinese University of Hong Kong, Hong Kong SAR, China; 2 Department of Ophthalmology, Beijing Friendship Hospital, Capital Medical University, Beijing, China; 3 Beijing Institute of Ophthalmology, Beijing Tongren Eye Center, Beijing Tongren Hospital, Beijing Key Laboratory of Intelligent Diagnosis Technology and Equipment for Optic Nerve-Related Eye Diseases, Capital Medical University, Beijing, China; 4 Department of Radiology, Emory University, Atlanta, GA USA; 5 Tsinghua Medicine, Tsinghua University, Beijing, China; 6 Beijing Tsinghua Changgang Hospital, Beijing, China; 7 Singapore Eye Research Institute, Singapore National Eye Centre, Singapore, Singapore; 8 Beijing Institute of Brain Disorders, Capital Medical University, Beijing, China; 9 Department of Neurosurgery, Xuanwu Hospital of Capital Medical University, Beijing, China; 10 Beijing Institute of Ophthalmology, Beijing Tongren Hospital, Capital Medical University, Beijing, China

**Keywords:** Evolution, Health care

## Abstract

Successfully adapting to life in the highest altitudes (“Roof of the world”) is a heritage of evolutionary adaptation for humans. With rising interest in adventure travel and expanding transport networks that facilitate mobility from low to high altitudes, provision of healthcare for populations living in high-altitude regions has re-emerged as an area of interest and research. These populations have several unique characteristics that limit the simple generalization of medical knowledge. First, these populations are naturally segregated into distinct ethnic groups, representing a unique marginal demographic. Second, the harsh natural environment, underdeveloped healthcare infrastructure, and limited research and understanding of healthcare needs, issues and challenges experienced by highland communities pose significant barriers to equitable healthcare access. The use of medical artificial intelligence and digital technology provides an opportunity to provide innovative solutions for these populations. However, these technologies would not facilitate health equity in their current state today as most are narrow in their application, are not trained on data representative of these regions, and ignore the multifactorial nature of being healthy that combines biological and physiological factors, in addition to environmental and socio factors. The success of generalist models for tasks such as scientific discovery provides a mechanism to leapfrog existing challenges and provide equitable care in these regions. In this paper, we discuss the opportunity of intelligent medical agents developed on generalist foundation models to meet the unique needs of high-altitude populations.

## Healthcare of populations living in high-altitude regions

Over 81.6 million people reside at altitudes above 2500 meters (8200 ft)^1^, commonly referred to as high-altitude populations. Unique physiological changes have been identified in these high-altitude communities from geographically isolated regions, including Tibet, the Andes, and Ethiopia, who represent one of the world’s most marginalized groups in terms of health equity^2,3^. These populations reveal unique heritable behavioral and genetic adaptations to their environment. Through prolonged residence and acclimatization, these individuals live in harmony with a low-oxygen, ultraviolet-intensive environment without significant impairment of physiological functions. Indeed, high-altitude populations represent an exceptional model for exploring the interplay between health conditions and geographical environment, particularly from the perspective of genetic evolution and molecular adaptation^4–6^. Notwithstanding their remarkable adaptability, high-altitude populations face substantial healthcare challenges^7^ (See Table 1) including limited access to comprehensive medical facilities, limited opportunities for medical education and resources^8^, shortage of healthcare professionals as a result of deficiencies in local training systems and difficulty in attracting and retaining qualified personnel, and weak infrastructure affecting tools for healthcare delivery. In addition, due to sparse healthcare facilities and limited interoperability, historical clinical documentation is fragmented and inconsistent, and notably deficient in standardized long-term follow-up medical data.

**Table 1:** Challenges of the current state of healthcare provision for high-altitude populations

Barriers to care	Modifiability by AI
Geographical isolation from advanced medical centers	Unchangeable
Extensive service radius of local healthcare facilities	Unchangeable
Higher reliability requirements for medical equipment	Unchangeable
Mountain sickness among short-term medical service personnel	Unchangeable
Inadequate basic medical infrastructure	Changeable
Limited medical education resources	Changeable
Lack of a scientifically sound and rational health monitoring system for high-altitude medicine	Changeable
Weak health awareness among original residents	Changeable
Limited investment attractiveness	Changeable

**Fig. 1:**
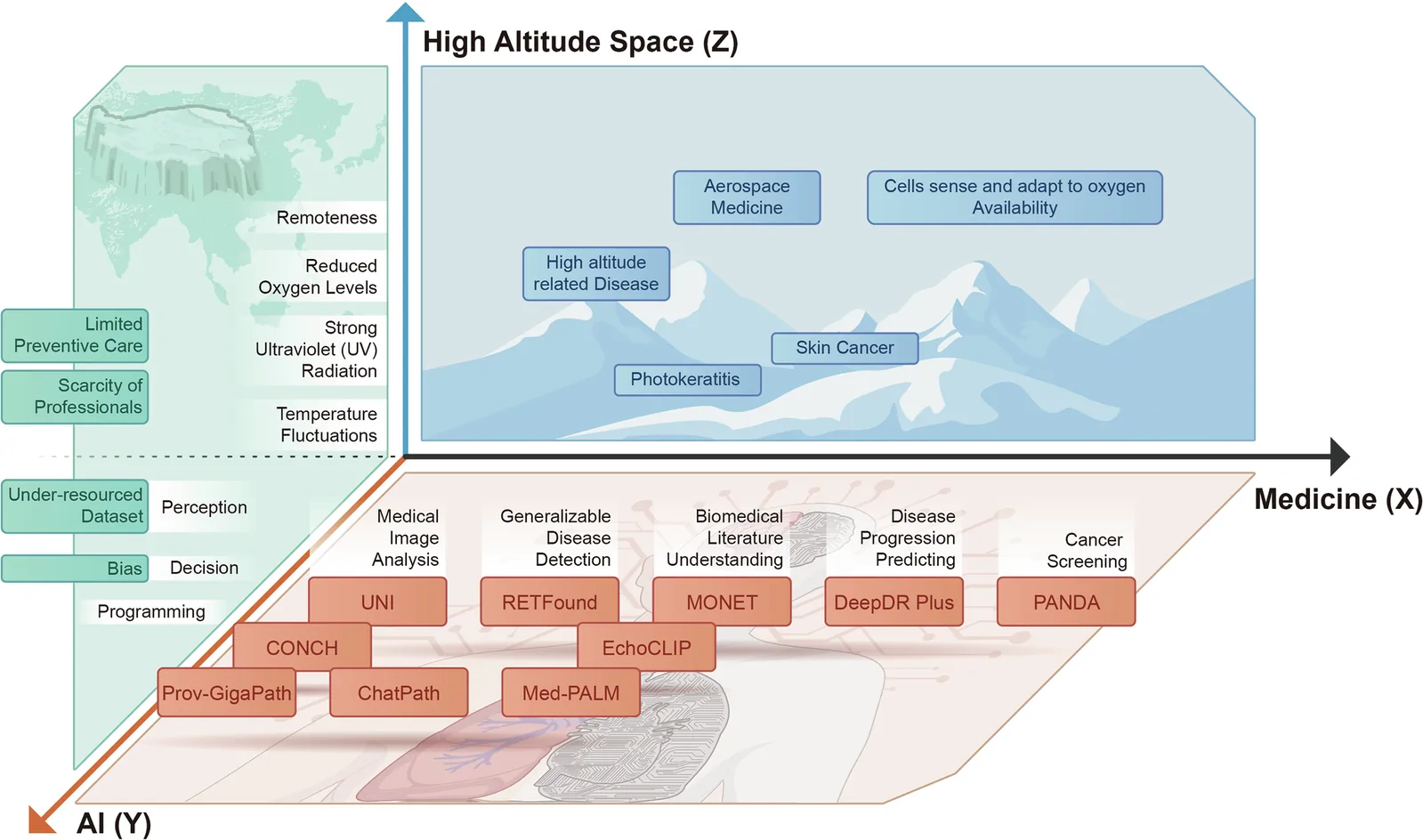
The “AI”, “Medicine”, and “High Altitude” axes form planes that do not intersect with each other. This conceptual framework depicts three largely independent domains: biomedical AI development, medical application landscapes, and the environmental and physiological challenges associated with high-altitude regions. While AI models increasingly support diverse clinical tasks, high-altitude environments introduce distinct disease patterns and healthcare constraints shaped by hypoxia, ultraviolet radiation, and geographical remoteness. Bridging these domains is essential for developing equitable and generalizable medical AI.

Compounding these challenges, the very factors that have left these communities underserved by traditional healthcare systems—geographical isolation, cultural distinctions, and limited resources—also significantly influence their interaction with and acceptance of emerging medical technologies. Populations in remote areas have lower perceptions of AI tools and digital literacy^9^. Both doctors and patients in remote high-altitude areas have lower acceptance of AI-in-loop diagnosis^10^. For example, in areas like the Qinghai-Tibet plateau, traditional Tibetan medicine holds greater significance for locals than contemporary medical practices^11^. This adds a layer of complexity to healthcare provision not observed in other regions of the world where trust plays a crucial role in deciding the acceptance of medical consultation by high-altitude communities. Specifically, medically sound treatments backed by evidence may be rejected by a patient in these regions when traditional medicine is available. While such information may be available to long term residents of these regions, short term visitors and personnel may be unaware of these options resulting in communication breakdown during patient management. AI tools hold promise to bridge such gaps when they are developed with community input, although this remains a lofty goal (See Fig. 1).

Despite these challenges in healthcare delivery and technology adoption, unique physiological characteristics of high-altitude populations offer remarkable opportunities for holistic health and medical advancement. Genetic adaptations of high-altitude populations present an unparalleled opportunity to elucidate human physiological resilience and evolutionary plasticity, encompassing a spectrum of modifications including hemoglobin regulation, pulmonary gas exchange efficiency, vascular reactivity, metabolic modulation, and antioxidant capacity. These adaptations provide a natural experiment that can inform medical research and healthcare innovation^4,12^. For example, in cardiopulmonary medicine, high-altitude adaptations could inform novel strategies for preventing and treating heart disease and respiratory disorders^13^. These adaptations include enhanced lung capacity and oxygen uptake efficiency, which could guide therapies for conditions like chronic obstructive pulmonary disease and pulmonary hypertension^14^.

Metabolically, high-altitude populations exhibit distinctive adaptations in their pathophysiology^15^ (see Supplementary Table S1). Tibetan highlanders, for instance, demonstrate augmented nitric oxide synthesis known to improve blood flow and oxygen delivery^16^. They also show enhanced glucose metabolism, potentially contributing to lower diabetes prevalence despite comparable BMI to lowlanders^17^. These metabolic adaptations are underpinned by genetic variations, particularly in EGLN1 and EPAS1 genes involved in the hypoxia-inducible factor (HIF) pathway^4^. Recent research has further informed the role of gut microbiota in metabolic adaptation to high-altitude regions. A meta-analysis of Qinghai-Tibet Plateau populations showed that microbiota-derived butyrate enhances energy metabolism while downregulating HIF-1α, potentially contributing to the unique metabolic adaptation to chronic hypoxia^18^. Furthermore, increased antioxidant defenses such as elevated superoxide dismutase activity in Tibetan highlanders may contribute to their adaptation to chronic hypoxia^19^. These findings have significant implications for gerontological research and interventions for age-related pathologies, given the critical role of oxidative stress in the aging process.

Additionally, medical knowledge derived from high-altitude environments extends beyond clinical applications. In sports medicine, it has inspired the “live high, train low” protocol, widely adopted by world-class athletes to optimize endurance performance. For example, distance runners and cyclists often use this method, spending several weeks living at high altitudes (typically 2000–2500 meters) while conducting intense training sessions at lower elevations to enhance their oxygen-carrying capacity and overall endurance^20^.

The integration of high-altitude medical insights with cutting-edge technologies, particularly artificial intelligence (AI), presents a transformative opportunity to bridge healthcare disparities between highland and lowland populations. AI-driven systems, including machine learning algorithms and predictive models, can facilitate personalized wellness strategies, enhance telemedicine capabilities, and catalyze cross-regional medical research collaborations.

## From AI agents to medical spatial intelligent agents for high-altitude populations

AI is increasingly applied to multiple medical domains including disease detection (RETFound^21^, Prov-GigaPath^22^), cancer screening (PANDA^23^), predicting disease progression (DeepDR Plus^24^), drug discovery^25^ and medical knowledge retrieval^26–29^. Despite extensive enthusiasm on the promise of AI to tackle several challenges in medicine, current models that haven’t been trained and tested in diverse geographical settings are unlikely to be useful for high-altitude populations^30–33^. This is because the introduction of a clinical case originating from a high-altitude region represents an out-of-distribution (OOD) scenario due to unique pathological and morphological features influenced by high-altitude environments or prevalent in ethnic minority populations. These features may diverge substantially from in-domain data upon which most AI systems are trained, as exemplified by conditions such as high-altitude heart disease (where right ventricular enlargement is typical rather than left ventricular enlargement found in lowlanders).

Lack of standard medical devices and infrastructure limitations makes it challenging to develop and deploy AI in highland populations. Current models require large amounts of training data, with an additional complexity of class imbalance in disease distribution and balance of normal versus abnormal findings observed in medical datasets. Even if data from high-altitude regions can be collected for AI training, their inclusion in training datasets does not guarantee equitable outcomes^30^ as these data are easily diluted from strategies to handle class imbalance, application of dropout during training and anonymization techniques like differential privacy where the minority class is oversampled or removed^34^. These data scarcity coupled with challenges inherent in their collection and lack of inclusion in model validation could instigate a pernicious cycle of “data-bias”^35^. Moreover, high-altitude medicine faces multifaceted challenges rooted in “spatial” dimensions. These comprise both axial factors, such as remoteness from economic and political hubs, and vertical factors, primarily the physiological and health implications of extreme elevations (see Fig. 1). Notwithstanding these obstacles, the scientific community continues to make significant strides in addressing existing knowledge gaps and developing preventive and therapeutic approaches in high-altitude medicine^4^, yet their dissemination may not be immediate to practitioners in these areas.

**Fig. 2:**
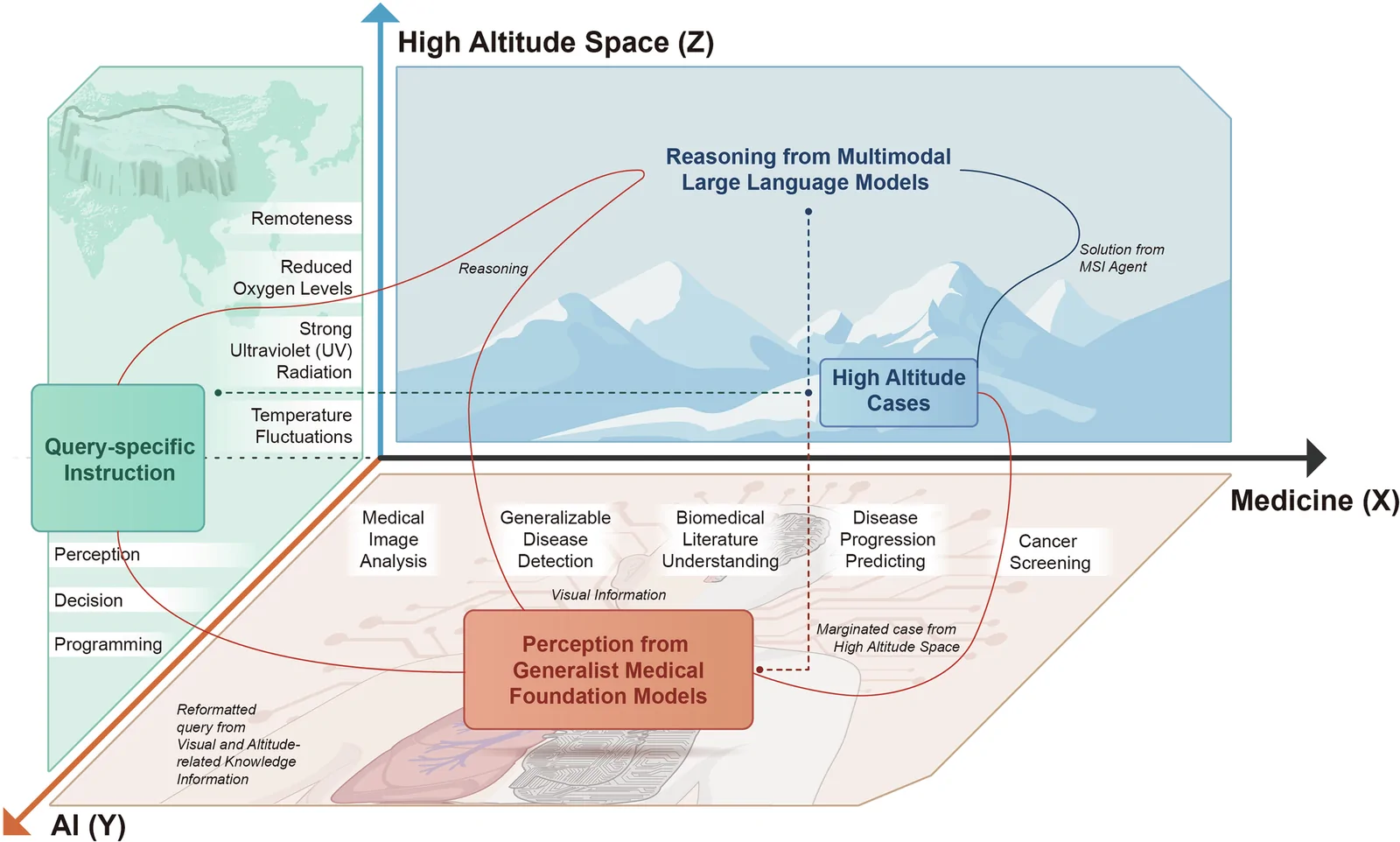
MSI is an agent for decoupling and accessing the capabilities of large AI models. It comprises two distinct stages: (1) Perception stage that first instructs GMAIs to extract and express visual information of the medical image; (2) Querying stage takes the altitude-related information into considerations via medical instruments and knowledge base. (3) Reasoning stage that utilizes an external LLM (GPT-4V, GPT-4o, etc) to conduct reasoning and answer the question based on the information from mentioned stage. Reader guide: Key elements required to interpret in this Figure operationally—MSI definition and boundaries (Box 1), evaluation and benchmark recommendations (Box 2), and governance/sovereignty mechanisms (Box 3)—are summarized below. Illustration created by a professional scientific illustration service commissioned by the authors.

The introduction of transformers and rapid development and adoption of multimodal foundation models that power various applications including ChatGPT have triggered a new wave of optimism about the role of AI in medicine. AI Agents refer to an autonomous entity with the ability to reflect, plan tasks, use tools and collaborate to achieve a goal. AI agents present a pathway that can allow AI models to fully play to their strengths and circumvent their weaknesses. Leveraging such AI agents in medicine can significantly impact global health equity for populations residing in remote high-altitude locations, distanced from sophisticated medical facilities and underrepresented in typical medical knowledge. Agents have the potential to facilitate enhanced perception, informed action, and intelligent evolution, potentially surmounting the constraints posed by the “roof of the world”, which is key to bridging the disparity between mainstream and marginalized communities residing in high-altitude regions (See Fig. 2 and Box 1). We refer to these agents as medical spatial intelligent agents (MSI agents) which contain a collection of “medical expert” foundation models and a general-purpose large language model as an orchestrator (Copilot) to route inquiries to the best solution to accomplish a multitude of tasks. The robust reasoning abilities of MSI agents enables them to extrapolate from their broad knowledge base to novel, OOD scenarios which are typical for high-altitude regions (see Box 1 for scope/non-goals; Box 2 for evaluation implications).

We envision that these MSI agents could be developed on multiple medical foundation models and medical instruments (e.g., medical robotics, unmanned aerial vehicle, weather API) with ability to perceive the surrounding environment and medical inputs^36^ (including data from biosensors, genetic, epigenetic, proteomic, microbiome, metabolomic, imaging, text, clinical, social determinants and environmental data), coupled with the ability of lifelong learning. Their ability could be tailored to provide synergistic collaboration between agents and medical professionals. Take high-altitude pulmonary edema (HAPE) as an example, which is a complex and multifactorial disease caused by excessive hypoxic pulmonary vasoconstriction due to low oxygen partial pressure at high altitudes. This increases the pulmonary artery pressure causing fluid leakage into the lungs. Damage to pulmonary capillary endothelial cells leads to increased permeability, allowing plasma proteins and fluid to seep out. Inflammatory responses triggered by the high-altitude environment release inflammatory mediators that further damage the endothelium. Additionally, genetic factors play a role, as certain gene variations can predispose individuals to HAPE. Traditional medical AI, trained primarily on low-altitude population data, might misinterpret HAPE’s characteristic radiographic findings and misclassify the patchy, peripheral edema distribution as atypical pneumonia, overlooking altitude-specific etiology. In the case of HAPE, MSI agents could integrate knowledge from diverse domains such as high-altitude physiology, genetic adaptations of highland populations, and radiological patterns specific to altitude-related illnesses. This holistic approach could lead to a more accurate interpretation of the radiographic findings, recognition of the altitude-specific context, and consideration of the patient’s genetic background, leading to a more accurate diagnosis. The MSI agents might also suggest altitude-specific treatment protocols, such as immediate descent or the use of portable hyperbaric chambers, which may not be part of standard low-altitude medical protocols. Establishing an initial proof-of-concept requires deploying MSI agents for a discrete clinical application, such as decision support for suspected HAPE in regional high-altitude clinics. Operating within a strictly constrained workflow, this pilot integrates local clinical observations, portable imaging or physiological measurements where available, and the retrieval of altitude-specific guidelines. These inputs generate structured recommendations complete with explicit uncertainty estimates and traceable evidence links (Box 1). Rather than optimizing exclusively for model generalization, the evaluation framework prioritizes safety, interpretability, and practical feasibility under real-world conditions, applying the component-level and equity-oriented criteria (see Box 2). Restricting the initial scope in this manner enables the rigorous validation of data pathways, auditability, and governance structures prior to scaling the technology for broader disease management. The following section summarizes specific benefits of MSI agents and their challenges to promote global health equity in high-altitude regions.

Box 1 MSI agents: definition, scope, and non-goals

- **Definition:** MSI agents are an **orchestration framework** that integrates (i) multimodal perception modules, (ii) context-aware retrieval and querying, and (iii) reasoning and orchestration, to produce **auditable, context-aware** clinical outputs for high-altitude settings.
- **Core modules (interfaces):**

1. **Perception layer** → Inputs: [X]; Outputs: [structured findings + uncertainty/OOD flags]
2. **Query layer** → Inputs: [findings + context]; Outputs: [altitude-/site-specific evidence + provenance]
3. **Reasoning layer** → Inputs: [findings + retrieved evidence]; Outputs: [recommendation + rationale + trace]
4. **Oversight layer** → Inputs: [logs + drift signals]; Outputs: [audit reports + escalation/decommission triggers]

- **Non-goals:**

1. Not a claim of fully autonomous diagnosis or treatment.
2. Not a guarantee to “solve bias”; rather, it **enables measurement and mitigation** via auditability.

Not dependent on any single foundation model; modules can be swapped based on resources and governance.

Box 2 Evaluation blueprint for MSI agents in high-altitude settings

- **Component-level evaluation**

1. **Perception:** accuracy (e.g., AUROC, F1), calibration (e.g., Expected Calibration Error), and OOD detection (e.g., AUROC for in-/out-of-distribution separation)
2. **Query:** retrieval relevance (e.g., recall@k, precision@k), coverage of altitude-specific guidelines, and expert-rated relevance
3. **Reasoning:** decision consistency and traceability (e.g., agreement with expert decisions, evidence–trace alignment)

- **System-level evaluation**

1. Task success (e.g., triage correctness, decision accuracy)
2. Safety events (e.g., critical misclassification rate, unsafe recommendation rate)
3. Robustness (latency under bandwidth constraints, missing data tolerance)
4. Human-AI interaction (clinician override rate, time-to-decision)

- **Altitude knowledge incorporation tests:**

1. Counterfactual testing (altitude-aware vs altitude-agnostic inputs)
2. Retrieval-provenance audits (consistency of cited altitude-specific evidence)
3. Guideline concordance (agreement with altitude-specific protocols)

- **Equity** **+** **deployment feasibility:**

1. Subgroup performance gaps (e.g., across ethnicity, altitude level)
2. Access constraints (latency, cost, infrastructure)
3. Failure reporting completeness and audit trace availability

**Example evaluation protocol**

A pilot MSI agents for HAPE triage may be evaluated on a retrospective cohort from high-altitude clinics using a leave-one-site-out design. Perception modules are assessed using AUROC and calibration error, retrieval modules using recall@k against curated guideline sets, and system-level decisions against expert consensus labels. Safety is quantified via false-negative rates for critical conditions, while audit completeness is evaluated by verifying the presence of traceable intermediate outputs for all cases.

## Operationalization of MSI agents: module interfaces and information flow

To enable evaluation and governance in high-altitude settings, MSI agents are best understood as a modular orchestration architecture rather than a monolithic end-to-end model (Table 2). In a typical workflow, a perception layer transforms raw clinical inputs (e.g., images, vitals, structured history) into intermediate structured findings with uncertainty and OOD flags. A query layer retrieves altitude- and site-specific information (e.g., local guidelines, epidemiological priors, logistics constraints, and validated physiological knowledge) with provenance. A reasoning layer integrates intermediate findings and retrieved evidence to generate a context-aware output (e.g., triage recommendation or decision support), accompanied by traceable evidence links and explicit uncertainty. Finally, an oversight layer defines logging, auditing, escalation, and decommissioning procedures to ensure accountability in deployment. This interface-based framing is intended to make MSI agents evaluable and governable in settings where both distribution shift and resource constraints limit traditional validation pathways (Boxes 1–3).

**Table 2:** MSI agents’ modules, interfaces, and audit fields

Module	Inputs	Outputs	Typical failure modes	Required audit fields
Perception	[images/vitals/history]	[structured findings + uncertainty + OOD flags]	low-quality input, domain shift, spurious features	input quality score, model version, uncertainty, OOD signal
Query	[findings + context]	retrieved evidence + provenance	retrieval misses local guidance, outdated sources	retrieved source IDs, timestamps, relevance score
Reasoning	findings + retrieved evidence	recommendation + rationale + trace	hallucination, over-reliance on priors	trace links, confidence calibration method, constraints applied
Oversight	logs + drift signals	audit report + escalation	silent degradation, governance gaps	incident reports, drift metrics, decommission triggers

To further clarify the operational feasibility of MSI agents, we provide a concrete instantiation using suspected HAPE as an example. In a typical deployment, a patient presenting with dyspnea and hypoxia at a high-altitude clinic could first undergo data acquisition including vital signs (SpO₂, heart rate), clinical history, and, where available, portable chest imaging (e.g., ultrasound or X-ray). The perception module processes these inputs using medical foundation models, including vision-based models (e.g., self-supervised or multimodal pretrained encoders) and vision–language models capable of aligning imaging findings with clinical semantics. These models transform raw inputs into structured intermediate representations, such as “bilateral patchy infiltrates” or “severity of hypoxemia,” accompanied by calibrated uncertainty estimates.

These intermediate outputs are then passed to the query module, which retrieves altitude-specific knowledge from curated sources, including high-altitude clinical guidelines, epidemiological priors, and environmental context (e.g., current altitude, rate of ascent, weather conditions). Retrieval outputs include ranked evidence with provenance metadata (e.g., guideline ID, timestamp, relevance score).

The reasoning module integrates structured findings and retrieved evidence using a general-purpose large language model with constrained prompting, producing a context-aware recommendation such as “suspected HAPE—recommend immediate descent and oxygen therapy,” accompanied by an explicit reasoning trace linking clinical findings to retrieved altitude-specific evidence.

Finally, the oversight module logs all intermediate artifacts (inputs, model outputs, retrieval sources, reasoning trace, uncertainty), performs consistency checks, and triggers escalation if predefined safety thresholds are exceeded (e.g., high uncertainty or conflicting evidence). This stepwise pipeline illustrates how MSI agents can be instantiated using existing model classes while maintaining auditability and modular extensibility.

Box 3 Governance and sovereignty “stack” (audit, oversight, decommissioning)

- **Community oversight:** co-developed acceptable-risk thresholds; approval rights for updates; ability to pause deployment
- **Auditing:** required logs (inputs, intermediate artifacts, retrieval sources, uncertainty, outputs); periodic review cadence
- **Decommissioning triggers:** drift, safety incidents, audit failure, governance violations

**What differs in high-altitude settings:** higher OOD risk + limited specialist coverage + stronger sovereignty constraints → governance must be integrated into system architecture, not only policy.

## Opportunities for MSI agents for high-altitude populations

### Medical education agent

MSI agents can serve as medical educators by leveraging their ability to extract and disseminate high-altitude knowledge related to medicine. Each agent can be assigned to different roles including a general domain specialist, an altitude medicine specialist and a researcher who verifies the literature and communicates comprehensive medical knowledge and surgical skills with students. These agents could be designed to interact with each other by simulating multidisciplinary discussions, as well as supporting telemedicine applications where upskilling is necessary for healthcare providers from developed regions who may also lack the requisite knowledge of high-altitude medicine.

### Epidemiological investigation agent

MSI agents can explore the distribution and variable presentation of health conditions in high-altitude regions^37^. An epidemiological investigation typically involves several sequential steps from initial case identification to public health communication. While traditional approaches follow these steps sequentially, MSI agents can strategically anticipate and plan for multiple phases simultaneously. This advanced foresight could allow MSI agents to optimize resource allocation, predict potential challenges, and adapt more efficiently than conventional methods, ultimately leading to smarter, more effective epidemiological responses. In sparse geographical regions typical of high-altitude regions, the scope of medical services is expansive and necessitates consideration of multiple data including the exposome of residents (oxygen content in the air, UV content, etc.), social determinants, data from wearable devices^38^, home cameras^39^ and long-term medical records. Multimodal MSI agents could aggregate these diverse data sources and summarize them to generate insights to support medical decision making.

### Scientific research agent

Observations of lower mortality rate from cardiovascular diseases^40^ and the existence of a dose-response relationship where an increase in elevation correlates with a decrease in obesity prevalence^33^ highlight the opportunity to generate novel medical insights that can impact chronic disease management. Today, this information is already translated to sports medicine with an opportunity to expand to other areas including drug discovery guided by adaptability from microscopic to special environments, genomic analysis^41^, disease progression prediction, and so forth. AI-driven autonomous experimentation offers transformative approaches to addressing high-altitude health disparities. Stanford University has advanced virtual human cell models^42^ while robotic laboratory systems have rapidly evolved across multiple research centers^43,44^. These developments have established several technological pillars: automated AI-aided experimental hardware^45^, Bayesian optimization-driven parameter tuning models^46^, and knowledge graphs that integrate millions of scientific relationships^47^. In high-altitude medicine, Sun et al.^48^ developed CPHNet, significantly enhancing HAPE compound screening efficiency, identifying two promising natural products- ferulic acid and resveratrol -with validation success rates exceeding 67%. The platform integrates advanced imaging algorithms with Cell Painting technology for comprehensive phenotypic analysis, validating findings through both 3D tissue models and animal studies^48^. MSI agents represent the next evolution in this technological progression. These agents may be capable of orchestrating these AI-aided automated experimental systems while aggregating multimodal data, correlating findings with scientific literature and generating testable hypotheses with corresponding study plans. There are multiple initiatives that are already attempting to accelerate scientific discovery using large language models and multimodal AI capabilities that can easily meet these gaps^39,49^.

### Psychological counseling agent

Mental health needs of communities living in high altitudes are critical due to harsh environmental surroundings characterized by extreme weather changes that compound barriers to service access as it is challenging to serve marginalized groups within a broad medical radius. MSI agents can play the role of a mental health counselor effectively^50^, especially in the face of diverse cultural, religious, and linguistic scenarios. The can provide diversified information, assist in stress management techniques, and further discern the requisite emotional values of these users and integrate them within the communicative process and monitoring^51^.

### Intelligent medical robots and devices

The promotion of mutual opportunities between MSI agents and hardware technology-oriented initiatives has emerged in underserved regions such as pastoral and mountainous areas. These durable robots, suitable for long-term deployment in highlands, continuously collect data, analyze epidemiological conditions, and provide tailored health guidance to meet community-specific needs. They could supplement human performance, for instance in cases where short-term professionals experience acute mountain sickness affecting their ability to function at their baseline (e.g., perform long surgeries). MSI agent-based embodied robots could circumvent such gaps i including shortages of advanced imaging equipment, which may be partially mitigated by generative AI-enabled tools^52,53^.

## Challenges and outlook

### Validation and verification

MSI agents are uniquely difficult to validate, owing to their unprecedented execution mode. We therefore propose an evaluation blueprint with component-level, system-level, and equity-level criteria (Box 2). At present, foundation models or intelligent agents are developed on mainstream populations excluding high-altitude populations, so MSI agents need to be validated on high-altitude scenarios before being deployed. Due to their generalist nature, MSI agents may adapt to high-altitude populations without task- or setting-specific development making it challenging to anticipate and test all their failure modes. Moreover, MSI agents handle complex inputs and outputs via multiple inferences for decision-making, making it extremely hard for clinicians to determine correctness of outputs. Consider, for instance, the task of examination of clinical records pertaining to high-altitude epidemiological investigations. MSI agents could potentially utilize medical foundation models for diagnosis^54^ and a large language model for interpretation and record summarization^55^. However, these models may overlook unique characteristics associated with the high-altitude perspective and be affected by AI hallucinations^56,57^, leading to summaries that deviate significantly from previous investigations. In such scenarios, a solitary clinician (not uncommon in these remote regions) may be unable to validate the accuracy of the MSI agents’ outputs.

Therefore, developers should conduct thorough validation and simplify the process of verifying AI agents’ outputs in the medical domain, ensuring no harm is inflicted upon underrepresented populations residing at high altitudes^58^. Additionally, incorporation of uncertainty can support medical decision making when using these agents because there exist different causative factors for a single disease, and identical treatments may not be universally effective. Beyond technical validity, MSI deployment in high-altitude settings also raises sociotechnical questions (particularly regarding data sovereignty, community governance, and benefit-sharing) that shape whether these systems are legitimate and equitable in practice (see Box 3).

### Bias

Neglecting data from high-altitude populations when training medical AI agents could be a significant oversight, resulting in a missed opportunity to develop helpful agents. Such agents must be developed considering unique geographical environments and cultural diversity of high-altitude populations that may affect agent deployment and use. While MSI agents show promise in overcoming geographical and resource barriers in high-altitude healthcare, they face significant challenges including data sovereignty concerns, algorithmic biases across ethnic populations, and hardware reliability issues in extreme environments.

Importantly, equity in medical AI should not be reduced to merely including under-represented high-altitude communities into mainstream datasets or optimizing models to better fit them. A more substantive notion of equity concerns who defines clinical priorities, who governs data use, who controls model updates and deployment, and who shares in the benefits generated by these systems. We therefore treat data sovereignty and indigenous knowledge not as peripheral “bias factors,” but as foundational design constraints for MSI agents: the goal is not simply to model marginalized communities more accurately, but to enable community agency in specifying objectives, governing model lifecycles, and determining acceptable-risk thresholds aligned with local values and health priorities.

Consistent with the framing of transforming communities from “research subjects to research agents,” MSI agents should from technologies developed for communities to systems co-developed with communities, to systems co-developed with communities, and—where feasible—to models and governance structures that communities can meaningfully steward. Practically, this implies participatory problem formulation (e.g., defining target outcomes beyond global AUC), community-governed data stewardship and consent, transparent logging and auditability, and local decision rights over deployment, updates, and decommissioning (see Box 3). Evaluation should likewise reflect community-relevant outcomes (e.g., missed diagnosis rates, access, feasibility, and health impact) rather than solely paper-oriented metrics.

We propose developing advanced MSI agents that integrate indigenous knowledge alongside data sovereignty and distributed laboratory networks—potentially transforming historically underrepresented communities from research subjects to research agents. These governance structures are intended to be established prior to model development through community partnership and shared decision-making, thereby shaping data collection, system objectives, and acceptable deployment boundaries from the outset (Box 3). Success hinges on addressing power imbalances and ensuring equitable benefit-sharing while preserving unique physiological variations, determining whether these technologies genuinely address or perpetuate health inequities. Additionally, trust is paramount. Disregarding religious beliefs and customs could undermine agent deployment effectiveness. This reflective step is often overlooked by both traditional AI developers and medical care volunteers. Human oversight is needed for agent supervision, but in cases where multiple agents are deployed there may be inadequate capital to provide this supervision necessitating deployment of an additional agent to act as an auditor of the AI’s output. Human oversight remains essential; however, in resource-constrained settings, continuous supervision may be infeasible. We therefore propose an auditable deployment design with automated monitoring and periodic human review, supported by explicit escalation and decommissioning criteria (Box 3).

### Social cognition

Besides infrastructure limitation and limited datasets, the greatest challenge to MSI agents success lies in nurturing the appropriate mindset of stakeholders including doctors, researchers, government and investors. Adequate discourse spanning the AI lifecycle is necessary to guide development considerations as well as improve understanding which can make it easy to adapt these technologies. We envision multiple agents used by different users. For example, physicians can harness these AI agents as indispensable adjuncts for patient data management, diagnostic suggestions, and facilitation of telemedicine services. From a research perspective, the wealth of data amassed by these agents can steer investigative efforts and aid in the formulation of more efficacious treatment modalities. On a governmental level, the deployment of these agents can be a catalyst for achieving healthcare parity, enhancing public health metrics, and alleviating the burden on existing healthcare infrastructures. The needs of these various stakeholders need to be prioritized to prevent conflict due to competing priorities. Some form of governance needs to be developed to ensure that agents are helpful to promote health equity, including decisions on when to retire agents that are harmful or unhelpful.

### Data infrastructure for addressing “under-resourced” datasets

Building datasets that meaningfully represent high-altitude populations requires collaborative, multi-site data initiatives rather than isolated institutional collections. We envision federated or consortium-based models in which regional hospitals, primary care sites, and public health programs contribute standardized data under shared governance agreements. Crucially, a minimum viable infrastructure must prioritize harmonized metadata schemas that capture dimensions typically absent from conventional medical datasets and general AI training corpora—specifically, precise altitude exposure, environmental context, and locally relevant clinical variables. By integrating these unique data elements with distributed quality-control protocols and privacy-preserving linkage mechanisms, such infrastructure can improve contextual completeness even when direct data sharing is infeasible. Such approaches aim not merely to increase dataset size, but to ensure contextual completeness and community governance, thereby avoiding the persistent “under-resourced dataset” problem observed in many global health AI efforts.

## Conclusion

In this paper, we discuss the uniqueness of populations living in high altitudes and the challenges they encounter in accessing healthcare. We further outline integrate various artifacts of these populations into a sociotechnical framework using multiple agents that integrate high-altitude acclimatization, individual susceptibility, cultural and social norms, with the objective of narrowing existing global health disparities for these subgroups. Our proposed multi-agent framework not only narrows the care gaps in these regions but also informs other populations by providing insights into anti-ischemic and anti-hypoxic mechanisms to develop alternative therapeutic options. For this to be successful, progress must be made not only in the technology approach but also by encouraging diversity and inclusivity in researchers from high altitudes^59^ to tackle deep-rooted social issues. Developing MSI agents involving high-altitude populations is a step towards global health equity in high-altitude populations.

## Supplementary information

Supplementary Material

## Data Availability

No datasets were generated or analyzed during the current study.
