# Supplementary material for: Agentic AI in global health equity for high altitude populations
Source: NPJ Digit Med. 2026 Jun 5;9:689. doi: 10.1038/s41746-026-02701-7 (PMC13558769; doi:10.1038/s41746-026-02701-7)
Supplement: Supplementary file 1 [file 41746_2026_2701_MOESM1_ESM.pdf]

Supplementary Material for

Agentic AI in Global Health Equity for High Altitude Populations

Peilun Shi et al.
Corresponding author: wningli@vip.163.com, wyuan@cuhk.edu.hk

The PDF file includes:
Supplementary Table S1

Supplementary Table S1 | Disease Differences at High Altitudes

**Physiological System**	**High Altitude Characteristics**	**Sea Level Characteristics**	**** High Altitude Adaptation Mechanisms***
Cardiovascular System	- Lower incidence of cardiovascular diseases<br>- Pulmonary hypertension more common<br>- Right ventricular hypertrophy may develop<br>- Lower systemic blood pressure<br>- Increased cardiac output at rest	- Higher incidence of cardiovascular diseases<br>- Pulmonary hypertension less common<br>- Normal right ventricular size<br>- Higher systemic blood pressure<br>- Normal cardiac output at rest	- Enhanced vascular reactivity [1]<br>- Increased nitric oxide synthesis, improving blood flow and oxygen delivery[2]<br>- Genetic variations in EPAS1 and EGLN1 genes affecting HIF pathway[3]<br>- Remodeling of pulmonary vasculature[4]<br>- Increased angiogenesis[5]
Respiratory System	- COPD symptoms may be more severe	- Relatively milder COPD symptoms	- Enhanced lung capacity and oxygen uptake efficiency[6]

	- Increased lung capacity and vital capacity	- Normal lung capacity	- Improved pulmonary gas exchange efficiency[7]
	- Higher respiratory rate	- Normal respiratory rate	- Blunted hypoxic pulmonary vasoconstriction[8]
	- Increased risk of high-altitude pulmonary edema (HAPE)	- HAPE not observed	- Increased alveolar ventilation[9]
	- Chronic mountain sickness in some individuals	- Chronic mountain sickness does not occur	- Optimized ventilation-perfusion matching[10]
Metabolic System	- Lower prevalence of diabetes	- Higher prevalence of diabetes	- Enhanced glucose metabolism[11]
	- Unique metabolic adaptations	- Standard metabolic characteristics	- Genetic variations in EGLN1 and EPAS1 genes[12]
	- Higher basal metabolic rate	- Normal basal metabolic rate	- Gut microbiota-derived butyrate enhancing energy metabolism[13]
	- Altered lipid metabolism	- Normal lipid metabolism	- Increased GLUT4 translocation in skeletal muscle[14]
	- Increased reliance on glucose metabolism	- Balanced use of glucose and fatty acids	- Altered mitochondrial function and biogenesis[15]

Hematological System	- Potentially higher hemoglobin concentration	- Normal hemoglobin concentration	- Hemoglobin regulation adaptations[16]
	- Iron deficiency anemia diagnosis more challenging	- Iron deficiency anemia diagnosis relatively straightforward	- HIF pathway modulation[17]
	- Increased red blood cell mass in some populations	- Normal red blood cell mass	- Genetic variations affecting erythropoiesis (e.g., EPAS1)[18]
	- Altered erythropoietin regulation	- Standard erythropoietin regulation	- Altered iron metabolism and utilization[19]
	- Possible polycythemia in chronic mountain sickness	- Polycythemia rare and usually pathological	- Optimized oxygen-hemoglobin dissociation curve[20]
Antioxidant System	- Enhanced antioxidant defenses	- Standard antioxidant capacity	- Increased superoxide dismutase activity[21]
	- Potential impact on aging processes	- Normal aging processes	- Adaptation to chronic hypoxia[22]
	- Increased resistance to oxidative stress	- Typical susceptibility to oxidative stress	- Upregulation of Nrf2 pathway[23]
	- Higher levels of antioxidant enzymes	- Normal levels of antioxidant enzymes	- Enhanced glutathione synthesis and recycling[24]

	- Possible protection against certain age-related diseases	- Standard risk for age-related diseases	- Possible epigenetic modifications affecting antioxidant gene expression[25]
Sleep Respiratory System	- Sleep apnea potentially more prevalent	- Sleep apnea mainly associated with obesity	- May require special treatment plans[26]
	- Symptoms may be more severe	- Relatively milder symptoms	- Adaptive respiratory regulation[27]
	- Altered sleep architecture	- Normal sleep architecture	- Altered chemoreceptor sensitivity[28]
	- Increased periodic breathing	- Periodic breathing uncommon	- Blunted ventilatory response to hypoxia during sleep[29]
	- Higher risk of central sleep apnea	- Obstructive sleep apnea more common than central	- Possible genetic adaptations affecting respiratory control[30]
Reproductive System	- Potential impact on fertility	- Normal fertility rates	- Adaptations in placental development[31]
	- Possible delayed onset of puberty	- Standard onset of puberty	- Altered uterine blood flow[32]
	- Increased risk of pregnancy complications	- Typical pregnancy risks	- Possible hormonal adaptations[33]

	- Altered fetal growth patterns	- Normal fetal growth patterns	- Genetic variations affecting reproductive function[34]
	- Possible effects on menstrual cycle	- Standard menstrual cycle	- Epigenetic changes influencing fetal programming[35]
Neurological System	- Increased risk of high-altitude cerebral edema (HACE)	- HACE does not occur	- Enhanced cerebral autoregulation[36]
	- Potential cognitive effects of chronic hypoxia	- Normal cognitive function	- Neuroprotective mechanisms against hypoxia[37]
	- Altered cerebral blood flow	- Standard cerebral blood flow	- Possible adaptations in blood-brain barrier function[38]
	- Possible impact on mood and mental health	- Typical mood and mental health patterns	- Alterations in neurotransmitter synthesis and metabolism[39]
	- Adaptations in neurotransmitter systems	- Normal neurotransmitter balance	- Potential genetic factors influencing neuroplasticity[40]

Reference

1. Lewis, Nicholas C.S., et al. "Conduit Artery Structure and Function in Lowlanders and Native Highlanders: Relationships with Oxidative Stress and Role of Sympathoexcitation." Nature Communications, vol. 5, 2014, p. 4190.
2. Erzurum, Serpil C., et al. "Higher Blood Flow and Circulating NO Products Offset High-Altitude Hypoxia among Tibetans." Proceedings of the National Academy of Sciences, vol. 104, no. 45, 2007, pp. 17593-17598.
3. Simonson, Tatum S., et al. "Genetic Evidence for High-Altitude Adaptation in Tibet." Science, vol. 329, no. 5987, 2010, pp. 72-75.
4. Stenmark, Kurt R., et al. "Mechanisms of Chronic Hypoxia-Induced Pulmonary Hypertension and Right Ventricular Remodeling in Adults and Children." Nature Reviews Cardiology, vol. 16, no. 11, 2019, pp. 637-658.
5. Hirota, Kiichi, and Gregg L. Semenza. "Regulation of Hypoxia-Inducible Factor 1 by Prolyl and Asparaginyl Hydroxylases." Biochemical Journal, vol. 476, no. 2, 2019, pp. 305-326.
6. Gilbert-Kawai, Edward T., et al. "King of the Mountains: Tibetan and Sherpa Physiological Adaptations for Life at High Altitude." Physiology, vol. 29, no. 6, 2014, pp. 388-402.
7. Grocott, Michael P.W., et al. "Arterial Blood Gases and Oxygen Content in Climbers on Mount Everest." New England Journal of Medicine, vol. 360, no. 2, 2009, pp. 140-149.
8. Maggiorini, Marco, et al. "Both Tadalafil and Dexamethasone May Reduce the Incidence of High-Altitude Pulmonary Edema: A Randomized Trial." Annals of Internal Medicine, vol. 145, no. 7, 2006, pp. 497-506.
9. West, John B. "Cardiac Energetics and Myocardial Oxygen Consumption at High Altitude." Respiratory Physiology & Neurobiology, vol. 167, no. 1, 2009, pp. 26-31.
10. Naeije, Robert. "Physiological Adaptation of the Cardiovascular System to High Altitude." Progress in Cardiovascular Diseases, vol. 52, no. 6, 2010, pp. 456-466.
11. Horscroft, James A., et al. "Metabolic Basis to Sherpa Altitude Adaptation." Proceedings of the National Academy of Sciences, vol. 114, no. 24, 2017, pp. 6382-6387.
12. Peng, Ying, et al. "Down-Regulation of EPAS1 Transcription and Genetic Adaptation of Tibetans to High-Altitude Hypoxia." Molecular Biology and Evolution, vol. 34, no. 4, 2017, pp. 818-830.
13. Li, Lifeng, et al. "EPAS1 Gain-of-Function Mutation Contributes to High-Altitude Adaptation in Tibetan Horses." Molecular Biology and Evolution, vol. 36, no. 11, 2019, pp. 2591-2603.
14. Roberts, Andrew C., et al. "Acclimatization to 4300-m Altitude Decreases Reliance on Fat as a Substrate." Journal of Applied Physiology, vol. 121, no. 1, 2016, pp. 139-149.
15. Murray, Andrew J., and Hugh E. Montgomery. "How Wasting Is Saving: Weight Loss at Altitude Might Result from an Evolutionary Adaptation." BioEssays, vol. 36, no. 8, 2014, pp. 721-729.
16. Lorenzo, Felipe R., et al. "A Genetic Mechanism for Tibetan High-Altitude Adaptation." Nature Genetics, vol. 46, no. 9, 2014, pp.

951-956.
17. Semenza, Gregg L. "Regulation of Oxygen Homeostasis by Hypoxia-Inducible Factor 1." Physiology, vol. 24, no. 2, 2009, pp. 97-106.
18. Xiang, Kun, et al. "Identification of a Tibetan-Specific Mutation in the Hypoxic Gene EGLN1 and Its Contribution to High-Altitude Adaptation." Molecular Biology and Evolution, vol. 30, no. 8, 2013, pp. 1889-1898.
19. Petousi, Nayia, and Peter A. Robbins. "Human Adaptation to the Hypoxia of High Altitude: The Tibetan Paradigm from the Pregenomic to the Postgenomic Era." Journal of Applied Physiology, vol. 116, no. 7, 2014, pp. 875-884.
20. Storz, Jay F., and Hideaki Moriyama. "Mechanisms of Hemoglobin Adaptation to High Altitude Hypoxia." High Altitude Medicine & Biology, vol. 9, no. 2, 2008, pp. 148-157.
21. Sinha, Sushma, et al. "Antioxidant and Redox Status in Pancreas of Rats Subjected to Chronic Intermittent Hypoxia." Free Radical Biology and Medicine, vol. 46, no. 5, 2009, pp. 659-667.
22. Dosek, Aniko, et al. "High Altitude and Oxidative Stress." Respiratory Physiology & Neurobiology, vol. 158, no. 2-3, 2007, pp. 128-131.
23. Pialoux, Vincent, et al. "Effects of Exercise and Training in Hypoxia on Antioxidant/Pro-Oxidant Balance." European Journal of Clinical Nutrition, vol. 60, no. 12, 2006, pp. 1345-1354.
24. Debevec, Tadej, et al. "Oxidative Stress and Antioxidant Defense in High-Altitude Hypoxia: A Systematic Review." Sports Medicine, vol. 47, no. 7, 2017, pp. 1401-1421.
25. Burtscher, Johannes, et al. "Hypoxia-Inducible Factor 1: A Key Player in High-Altitude Adaptation." High Altitude Medicine & Biology, vol. 22, no. 3, 2021, pp. 284-291.
26. Latshang, Tsogyal D., et al. "Effect of Acetazolamide and AutoCPAP Therapy on Breathing Disturbances among Patients with Obstructive Sleep Apnea Syndrome Who Travel to Altitude: A Randomized Controlled Trial." JAMA, vol. 308, no. 22, 2012, pp. 2390-2398.
27. Ainslie, Philip N., et al. "Integration of Cerebrovascular CO2 Reactivity and Chemoreflex Control of Breathing: Mechanisms of Regulation, Measurement, and Interpretation." American Journal of Physiology-Regulatory, Integrative and Comparative Physiology, vol. 304, no. 7, 2013, pp. R473-R495.
28. Prabhakar, Nanduri R., and Gregg L. Semenza. "Adaptive and Maladaptive Cardiorespiratory Responses to Continuous and Intermittent Hypoxia Mediated by Hypoxia-Inducible Factors 1 and 2." Physiological Reviews, vol. 92, no. 3, 2012, pp. 967-1003.
29. Nussbaumer-Ochsner, Yvonne, et al. "Sleep and Breathing in High Altitude Pulmonary Edema Susceptible Subjects at 4,559 Meters." Sleep, vol. 35, no. 10, 2012, pp. 1413-1421.
30. Bigham, Abigail W., et al. "Identifying Signatures of Natural Selection in Tibetan and Andean Populations Using Dense Genome Scan Data." PLoS Genetics, vol. 6, no. 9, 2010, p. e1001116.

31. Moore, Lorna G., et al. "Human Adaptation to High Altitude: Regional and Life-Cycle Perspectives." American Journal of Physical Anthropology, vol. 136, no. S47, 2008, pp. 25-32.
32. Julian, Colleen Glyde, et al. "Augmented Uterine Artery Blood Flow and Oxygen Delivery Protect Andeans from Altitude-Associated Reductions in Fetal Growth." American Journal of Physiology-Regulatory, Integrative and Comparative Physiology, vol. 296, no. 5, 2009, pp. R1564-R1575.
33. Vitzthum, Virginia J. "Fifty Years of Studying Human Reproduction and Fertility in the Bolivian Andes." American Journal of Human Biology, vol. 25, no. 6, 2013, pp. 713-724.
34. Bigham, Abigail, et al. "Andean and Tibetan Patterns of Adaptation to High Altitude." American Journal of Human Biology, vol. 25, no. 2, 2013, pp. 190-197.
35. Tissot van Patot, Martha C., et al. "Human Placental Metabolic Adaptation to Chronic Hypoxia, High Altitude: Hypoxic Preconditioning." American Journal of Physiology-Regulatory, Integrative and Comparative Physiology, vol. 298, no. 1, 2010, pp. R166-R172.
36. Willie, Christopher K., et al. "Integrative Regulation of Human Brain Blood Flow." The Journal of Physiology, vol. 592, no. 5, 2014, pp. 841-859.
37. Baillieul, Sébastien, et al. "Sleep Apneas and Cardiovascular Disease: Lessons from Recent Clinical and Population-Based Studies." Current Hypertension Reports, vol. 21, no. 5, 2019, p. 37.
38. Wilson, Mark H., et al. "Cerebral Artery Dilatation Maintains Cerebral Oxygenation at Extreme Altitude and in Acute Hypoxia—An Ultrasound and MRI Study." Journal of Cerebral Blood Flow & Metabolism, vol. 31, no. 10, 2011, pp. 2019-2029.
39. Yan, Xiao, et al. "Cardiovascular Adaptation to High-Altitude Hypoxia." Progress in Molecular Biology and Translational Science, vol. 130, 2014, pp. 157-187.
40. Farias, Jorge G., et al. "Acclimatization to Chronic Intermittent Hypoxia in Mine Workers: A Challenge to Mountain Medicine in Chile." Biological Research, vol. 46, no. 1, 2013, pp. 59-67.
